# Changes in the Microbial Communities of *Picea schrenkiana* Needles Following *Lirula macrospora* Infection

**DOI:** 10.3390/plants15030449

**Published:** 2026-02-01

**Authors:** Saiyaremu Halifu, Sijia Zhang, Guorong Liu, Libin Yang, Xun Deng

**Affiliations:** 1 College of Urban and Environmental Sciences, Shihezi University, Shihezi 832003, China; 2 Institute of Nature and Ecology, Heilongjiang Academy of Sciences, Harbin 150036, China; 3 Institute of Forestry Protection, Heilongjiang Forestry Academy, Harbin 150040, China

**Keywords:** *Picea schrenkiana*, *Lirula macrospora*, needle blight, community succession, cross-kingdom network

## Abstract

*Picea schrenkiana* is a keystone species in Central Asian ecosystems currently threatened by climate-driven disease outbreaks. Here, we investigated the causal agent of needle blight and characterized the associated microbial dynamics. By integrating tissue isolation, Koch’s postulates, and high-throughput amplicon sequencing across a disease severity level, we confirmed *Lirula macrospora* as the etiological agent. Community analysis revealed that disease severity is the primary driver of succession, with alpha diversity peaks at the moderate infection stage. Notably, the abundance of *Lirula* surged from 2.56% in healthy needles to 65.10% in severe cases, displacing the core endophyte *Phaeococcomyces*, while potentially beneficial bacteria like *Sphingomonas* showed only transient enrichment. Furthermore, cross-kingdom co-occurrence network analysis revealed marked topological restructuring whereby the system reached a complex ecological “tipping point” during moderate stage before undergoing significant simplification. As the disease progressed, *L. macrospora* shifted from a peripheral node to a central hub, effectively dismantling the native microbial network. We conclude that *L. macrospora* infection triggers a cascading collapse of the needle microbiome, driving a phase shift from a healthy homeostasis to a pathogen-dominated state. These findings elucidate the critical mechanisms of pathogen-microbiome interactions and provide a theoretical basis for the ecological management of *P. schrenkiana* forests.

## 1. Introduction

*Picea schrenkiana* (Schrenk’s spruce), the dominant constructive species of the forest ecosystems in the Tianshan Mountains, is predominantly distributed along the northern slopes, accounting for approximately 54% of the forest area in Xinjiang. Situated at the critical ecotone between glaciers and deserts, this species intercepts and regulates mountain precipitation and glacial meltwater, thereby playing an irreplaceable role in regional water conservation, soil retention, and the maintenance of biodiversity [1,2]. However, *P. schrenkiana* is highly sensitive to environmental fluctuations. In recent years, factors such as rising mean annual temperatures, drought stress, declining groundwater tables, high stand density, low species diversity, and anthropogenic disturbances have collectively driven the decline of *P. schrenkiana* populations [3,4]. Concurrently, the incidence of pests and diseases related to the *P. schrenkiana* has increased annually, with needle blight emerging as the most severe threat in terms of occurrence and transmission intensity, posing significant risks to the stability and biodiversity of the Tianshan ecosystem.

Needle blight is a prevalent pathology in *Pinaceae*, typically manifesting as lesions, chlorosis, and necrosis, which can lead to tree mortality over perennial infections. Previous studies have characterized similar afflictions in spruce species. For instance, Li et al. [5] reported that *Picea asperata* (10–15 years old) exhibited symptoms such as needle yellowing and darkening at lesion interfaces, followed by withering and abscission, where the causal agents were identified as *Fusarium oxysporum* and *Fusarium avenaceum* through pathogen isolation. Similarly, Wang et al. [6] observed that the rust fungus *Chrysomyxa unger* causes needle and cone rust in spruce, primarily affecting natural and plantation forests in northwestern and southwestern China, resulting in substantial economic losses to the forestry industry. In the specific context of *P. schrenkiana* in Tianshan region, needle rust caused by *Chrysomyxa deformans* is a common disease that primarily damages current-year tender needles and shoots of *P. schrenkiana*. As the disease progresses, it causes the desiccation and death of young twigs and leaves, thereby severely impeding normal growth, accelerating tree decline, and predisposing the host to secondary pest and disease infestations [7]. More specific to the *Lirula genus*, Li et al. [8] reported that *Lirula macrospora* causes needle blight on *Abies georgei* in Yunnan, China, forming elliptical to linear lesions where necrotic needles characteristically remain attached to living branches. Similar symptoms—needle browning with prolonged retention—have been documented in North America on *Picea glauca* (White spruce) and *Picea pungens* (Colorado blue spruce) [9]. Although *L. macrospora* has been recorded both domestically and internationally, the specific micro-ecological mechanisms underlying its pathogenicity within the *P. schrenkiana* forests of the Tianshan Mountains remain unexplored.

Microorganisms play a pivotal role in maintaining the health and stability of plant ecosystems. Over 400 million years of co-evolution, host plants have established stable endophytic and epiphytic microbial communities [10]. Under specific biotic or abiotic stress, host plants actively regulate the assembly of their associated microbial communities to form a defensive barrier against pathogen invasion. However, pathogen intrusion often disrupts the stability of these symbiotic networks, leading to phyllosphere dysbiosis, which in turn accelerates disease progression [11,12]. According to the diversity-resistance hypothesis, communities with high diversity in the phyllosphere typically possess more complex interspecific interaction networks, allowing them to occupy ecological niches more effectively and competitively exclude pathogens [13]. As a core component of the host micro-ecosystem, endophytic fungi not only participate in host defense system construction through secondary metabolite secretion, niche competition, and induced systemic resistance but also their community stability and successional dynamics are key determinants of plant tolerance to biotic stresses [14]. For example, Li et al. [15] observed that in *Pinus koraiensis* infected by pine wood nematode, the community structure, diversity, richness, and functional gene composition of endophytes shifted significantly with increasing disease severity, indicating a robust microbiome response to pathogenic stress. Similarly, studies on cotton have shown that disease-resistant cultivars harbor more complex endophytic networks with higher core microbiome diversity compared to susceptible ones [16]. With the advent of high-throughput sequencing technology, amplicon sequencing has emerged as an efficient tool for microbial diversity analysis and has been widely applied to dissect the structure and dynamics of microbial communities during plant disease progression [17,18]. This technology enables high-resolution analysis of microbial communities in distinct host plant microecosystems, providing critical insights into the complex relationships between pathogen invasion, community succession, and host response, which is essential for elucidating pathogenicity mechanisms and micro-ecological changes.

Despite the recognition of endophytic microbial communities as the last line of defense for plants against pathogens, the mechanisms by which *P. schrenkiana* leaf blight pathogens breach this barrier and induce endophytic dysbiosis remain unclear. To address this gap, this study focuses on the needle endosphere micro-ecosystem of *P. schrenkiana* affected by needle blight. We first confirmed the etiological agent through field investigation and pathogen identification. Subsequently, strict surface sterilization was performed to exclude epiphytic microbial interference. Using ITS and 16S rRNA amplicon sequencing technologies, we accurately analyzed the reshaping effects of pathogen invasion on endophytic fungal and bacterial community structures, as well as the successional patterns of key symbiotic taxa, across a gradient from healthy to severely infected trees. This study aims to reveal the response patterns of major functional groups in endophytic microbial communities driven by *L. macrospora*, clarify the ecological mechanisms of dysbiosis during pathogenesis, and provide a scientific basis for exploring biocontrol-potential endophytic resources and formulating forest health management strategies.

## 2. Results

### 2.1. Symptom Observation and Pathogen Identification

**Figure 1:**
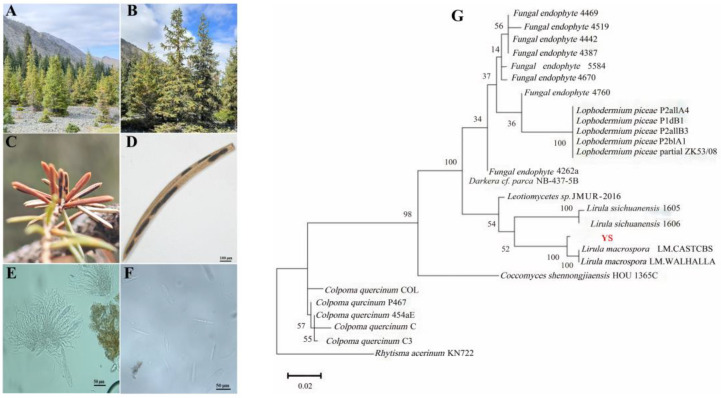
Disease symptom observation and pathogen identification. (**A**,**B**) A severely diseased *P. schrenkiana* tree. (**C**,**D**) Needles infected by *L. macrospora*. (**E**) Asci, ascospores, and paraphyses. (**F**) Close-up of asci and ascospores. (**G**) Maximum-likelihood phylogenetic tree based on concatenated ITS, AF462441, and 58S gene sequences, showing the phylogenetic position of the isolated pathogen (YS: *L. macrospora*).

Severely infected *P. schrenkiana* trees exhibited overall chlorosis and reduced vigor (Figure 1A,B). Lesions appeared on all needle surfaces, presenting as black-brown to glossy black, elliptical to linear structures, measuring 0.6–8 mm in length and 360–540 μm in width. In some cases, lesions extended longitudinally to cover nearly the entire needle. They were characterized by rounded or obtuse ends, slight elevation, and a faint, grayish-brown marginal line. Infected needles gradually turned brown but were not prematurely shed, remaining attached to living branches [8,19] (Figure 1C,D). The ascomata (fruiting bodies) were observed on the needle surface, revealing a greyish hymenium upon longitudinal splitting of the epidermis. The internal stroma consisted of textura intricata, 12–22 μm thick. Paraphyses were filiform, approximately the same length as the asci, 1.5–2.5 μm wide, and sometimes gradually swelling at the apex, and occasionally branched near the tip [20] (Figure 1E). The asci were clavate or cylindrical-clavate, measuring 90–125 × 13–16 μm, with a rounded or obtusely pointed apex and a short stalk, arranged in fascicles and each contained eight ascospores. The ascospores were colorless, clavate with rounded ends, and tapered slightly toward the base or both ends, measuring 50–68 × 2–3 μm (Figure 1F).

In pathogenicity tests, needles inoculated using wounding and non-wounding methods developed symptoms 60 days post-inoculation that were identical to those observed in the field. Microscopic examination of sections from these artificially infected needles revealed that the morphology of the pathogen was consistent with the field isolates. The infection incidence of wounded, heat-scarified, and non-wounded needles was 82%, 80%, and 40%, respectively. Molecular identification was performed by amplifying and sequencing the ITS, AF462441, and 58S gene regions. BLASTn analysis of the resulting sequences showed that the obtained sequences shared 99.41–100% identity with *L. macrospora* sequences in the NCBI database. The phylogenetic tree, constructed based on the concatenated sequences, showed that the isolated pathogen clustered within the *L. macrospora* clade with 100% bootstrap support (Figure 1G). Based on this combined morphological and molecular evidence, the causal agent was unequivocally identified as *L. macrospora*.

### 2.2. Microbial Diversity in Needles with Varying Disease Severity

**Figure 2:**
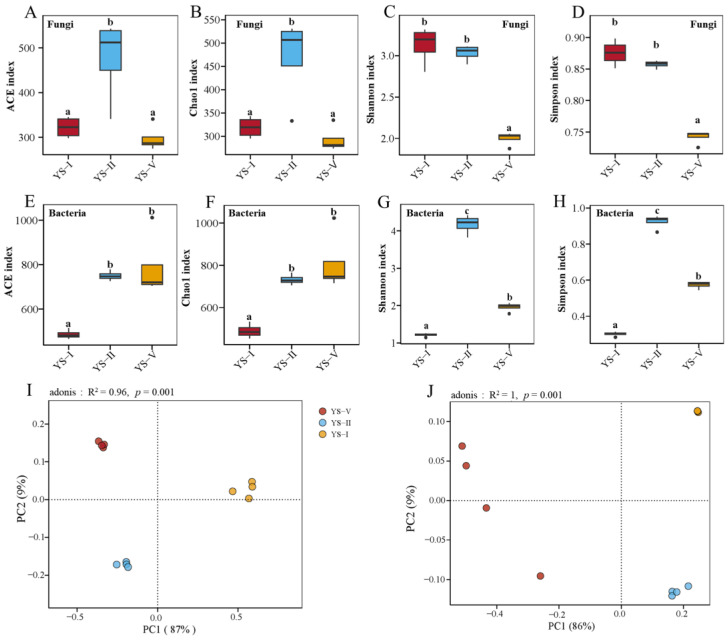
Diversity and structure of microbial communities in *P. schrenkiana* needles at different disease severity levels. (**A**–**D**) Comparison of fungal community alpha diversity indices (ACE, Chao1, Shannon, Simpson) among YS-I, YS-II, and YS-V groups. (**E**–**H**) Comparison of bacterial community alpha diversity indices among the three groups. Different lowercase letters (a, b, c) indicate significant differences at *p* < 0.05 (c > b > a). Principal Co-ordinates Analysis (PCoA) of fungal (**I**) and bacterial (**J**) communities based on Bray–Curtis dissimilarity.

To investigate the impact of *L. macrospora* infection on the needle microbiome, we analyzed the fungal and bacterial community structures in healthy (YS-I), moderately infected (YS-II), and severely infected (YS-V) needles by sequencing the ITS and 16S rRNA gene fragments, respectively. Sequences were clustered into Operational Taxonomic Units (OTUs) at 97% similarity. The fungal community exhibited a non-linear response to disease progression. Fungal species richness, as measured by ACE and Chao1 indices, was significantly higher in YS-II compared to both YS-I and YS-V needles (*p* < 0.05) (Figure 2A,B). Furthermore, community diversity and evenness, indicated by the Shannon index and Simpson’s diversity index, were significantly higher in YS-I and YS-II groups than in the YS-V group (Figure 2C,D). Specifically, both indices showed a consistent trend where diversity was maintained at a high level during moderate infection but declined significantly as the disease became severe.

In contrast, the bacterial community exhibited a different trend. Bacterial species richness (ACE and Chao1 indices) was significantly lower in the YS-I group compared to both the YS-II and YS-V groups (*p* < 0.05) (Figure 2E,F), suggesting that the onset of disease promotes an increase in bacterial richness. The Shannon index and Simpson’s diversity index revealed that bacterial diversity was highest in the YS-II group and lowest in the YS-I group, with the YS-V group at an intermediate level (Figure 2G,H). These findings suggest that as the disease progresses, bacterial richness increases, while diversity follows a unimodal pattern, first increasing and then declining. Principal Co-ordinates Analysis (PCoA) based on Bray–Curtis distances showed a clear separation of fungal communities among the YS-I, YS-II, and YS-V groups, with the first two axes explaining 96% of the total variance (Figure 2I). This separation was statistically significant, as confirmed by PERMANOVA (R^2^ = 0.96, *p* = 0.001), demonstrating a profound impact of the disease on fungal community structure. The bacterial communities also displayed an even more pronounced structural shift, with PCoA explaining 95% of the variation and complete separation of the three groups along the disease severity (Figure 2J). This clear separation was strongly supported by PERMANOVA (R^2^ = 1, *p* = 0.001), indicating that the bacterial community response is not merely a shift between healthy and diseased states but a structured, gradient-like change that intensifies with disease progression. These results demonstrate that pathogen infection profoundly restructures both fungal and bacterial communities by altering the needle microenvironment, with distinct response patterns observed for each microbial kingdom.

### 2.3. Microbial Community Composition and Differential Taxa

**Figure 3:**
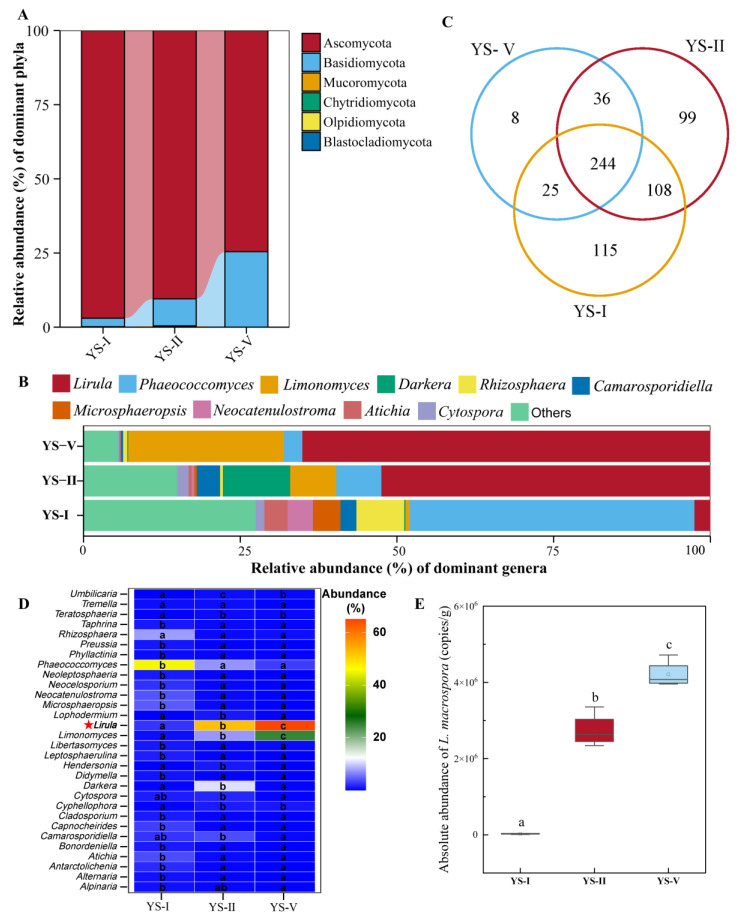
Fungal community composition, differential species, and pathogen abundance changes in needles across different infection severity levels. (**A**) Fungal community composition at the phylum level. (**B**) Fungal community composition at the genus level. (**C**) Venn diagram analysis of fungal communities. (**D**) Differential species heatmap analysis at the species level; (**E**) Absolute quantification analysis of *L. macrospora.* Different lowercase letters (a, b, c) indicate significant differences at *p* < 0.05 (c > b > a).

At the phylum level, Ascomycota and Basidiomycota were the dominant fungal phyla (Figure 3A). The relative abundance of Ascomycota was highest in YS-I (96.97%) and progressively decreased with increasing disease severity (90.46% in YS-II, 74.55% in YS-V). Conversely, the relative abundance of Basidiomycota steadily increased across the disease gradient (2.86%, 9.10%, and 25.44% in YS-I, YS-II, and YS-V, respectively). At the genus level (Figure 3B), the abundance of *Lirula*, the causal pathogen, dramatically increased with disease severity (2.56%, 52.49%, and 65.10%), confirming its expansion and eventual dominance within the microbial community of infected needles. In stark contrast, *Phaeococcomyces*, a potential endophyte, was the core genus in healthy needle community but was significantly displaced as the disease progressed (from 45.41% to 2.93%, *p* < 0.05). *Limonomyces* showed a positive response to disease progression, with significant enrichment in the late stages, suggesting a primarily saprotrophic lifestyle, wherein it colonizes necrotic tissues to utilize nutrients released following host cell death caused by *L. macrospora*. In contrast, the abundance of *Darkera* peaked at the moderate infection stage, implying that it exploited the “opportunity window” created by reduced host immunity or altered environmental conditions for proliferation. However, its subsequent decline indicates that *Darkera* was ultimately competitively excluded by the dominant pathogen *L. macrospora* during late-stage infection. Meanwhile, the abundance of *Rhizosphaera* decreased sharply with disease onset, reflecting high sensitivity to the biotic stress induced by *L. macrospora* invasion. Consequently, in this specific pathosystem, the dominant status of *Rhizosphaera* can be regarded as a biomarker of the asymptomatic stage, representing the baseline community structure before the outbreak of needle blight.

Venn diagram analysis revealed a core fungal community of 244 OTUs shared across all samples (Figure 3C). The number of unique fungal OTUs decreased with disease severity (115 in YS-I, 99 in YS-II, 8 in YS-V), suggesting that the disease imposes a strong selective pressure that reduces species specificity and the diversity of rare fungi. Differential abundance analysis (Figure 3D) confirmed that the relative abundance of *L. macrospora* was significantly elevated in diseased groups, where it became the dominant species (52.49% in YS-II, 65.10% in YS-V). This was corroborated by qPCR analysis (Figure 3E), which showed that the absolute abundance of *L. macrospora* was significantly elevated in infected groups, rising from 2.83 × 10^4^ copies in YS-I to 2.74 × 10^6^ in YS-II and 4.21 × 10^6^ copies in YS-V. This confirms *L. macrospora* as the dominant causal agent. The abundance of *Limonomyces* also increased dramatically with disease severity (from 0.69% to 24.84%), suggesting it may be involved in secondary infection or saprophytic processes. Conversely, *Phaeococcomyces* had the highest relative abundance (>45%) in healthy needles and was significantly depleted in diseased samples (*p* < 0.01), reinforcing its potential role as a key beneficial endosymbiont in healthy hosts.

**Figure 4:**
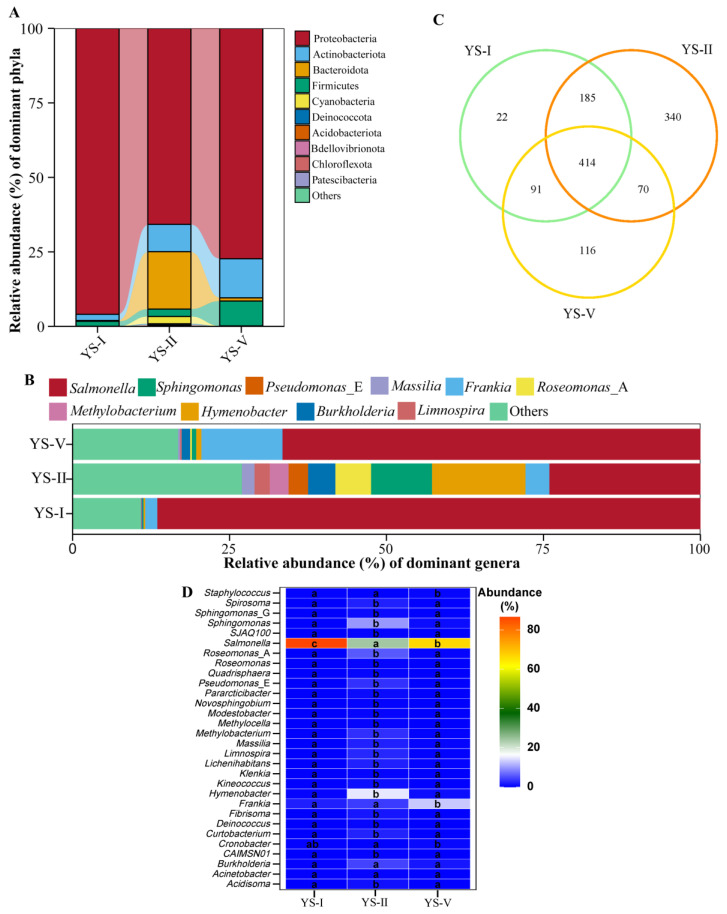
Bacterial community composition, differential species, and pathogen abundance changes in needles across different infection severity levels. (**A**) Bacterial community composition at the phylum level. (**B**) Bacterial community composition at the genus level. (**C**) Venn diagram analysis of Bacterial communities. (**D**) Differential species heatmap analysis at the species level. Different lowercase letters (a, b, c) indicate significant differences at *p* < 0.05 (c > b > a).

At the bacterial phylum level (Figure 4A), Proteobacteria was the dominant phylum across all groups but its relative abundance decreased with disease onset (95.95% in YS-I, 65.77% in YS-II, 77.30% in YS-V). In contrast, the abundances of Actinobacteriota and Firmicutes increased with disease severity, potentially linked to their antagonistic properties or competitive advantages. At the genus level (Figure 4B), Salmonella was the predominant genus, showing a distinct stage-specific response (86.51% in YS-I, 24.07% in YS-II, and 66.57% in YS-V). Genera such as *Frankia*, *Burkholderia*, and *Pseudomonas_E* were enriched in the diseased groups. Notably, several genera including *Sphingomonas*, *Methylobacterium*, *Massilia*, and *Limnospira* peaked during the moderately diseased stage (YS-II).

Venn diagram analysis for bacterial identified a core community of 414 OTUs (Figure 4C). The number of unique OTUs was highest in the YS-II group (340) compared to YS-I (22) and YS-V (116), indicating the most dynamic community response and enrichment of specific taxa occurs at the moderate infection stage. Differential abundance analysis (Figure 4D) revealed that *Salmonella* was the dominant genus (86.51% in YS-I, 24.08% in YS-II, and 66.59% in YS-V), with its abundance significantly decreasing during the initial infection but increasing in severely diseased needles. Multiple *Sphingomonas* OTUs were significantly enriched in the YS-II group. Additionally, the abundances of *Frankia* increased progressively with disease severity.

### 2.4. Pathogen Invasion Drives the Restructuring of the Needle Microbial Co-Occurrence Network

**Figure 5:**
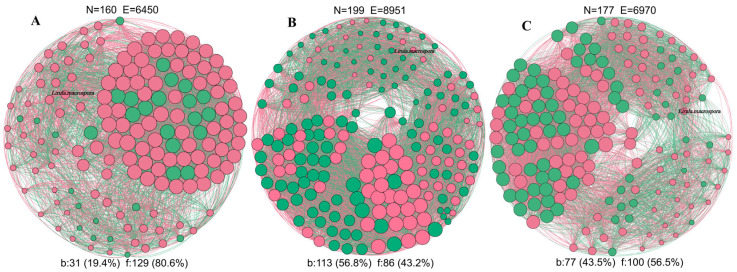
Cross-kingdom network analysis of fungal and bacterial communities in needles at different disease severity levels. Cross-kingdom networks for the (**A**) healthy (YS-I), (**B**) moderately infected (YS-II), and (**C**) severely infected (YS-V) groups. N: number of nodes; E: number of edges; the Latin name in the figure is *L. macrospora*; b represents bacteria (green circles) and f represents fungi (pink circles). Red lines indicate positive correlations, while green lines indicate negative correlations.

To explore impact of *L. macrospora* on microbial community interactions, we constructed cross-kingdom (bacteria-fungi) co-occurrence networks for YS-I, YS-II, and YS-V samples (Figure 5). The results revealed a significant restructuring of the community interaction patterns as the disease progressed. Network complexity, defined by the number of nodes and edges, increased sharply from the healthy stage (YS-I: 160 nodes, 6450 edges, including 3597 positive and 2853 negative correlations) and peaked at the moderate infection stage (YS-II: 199 nodes, 8951 edges, including 5094 positive and 3857 negative correlations), indicating “mobilization” of the microbiome in response to invasion. Subsequently, complexity significantly declined at the severe infection stage (YS-V: 177 nodes, 6970 edges, including 4187 positive and 2783 negative correlations). Analysis of interaction properties showed that although the absolute frequency of negative edges was highest in YS-II (3857), the global proportion of negative correlations decreased progressively from YS-I (44.23%) to YS-V (39.93%). Correspondingly, the ratio of positive to negative correlations steadily increased, reaching a maximum of 1.51 in YS-V, indicating a general community shift toward cooperative succession. The connectivity of the pathogen *L. macrospora* within the network changed significantly across disease stages. Its connectivity shifted from 37 edges (21 positive/16 negative) in healthy needles to 38 edges (15 positive/23 negative) in moderately infected needles. In the severely infected network, its total connections decreased to 18 (7 positive/11 negative).

## 3. Discussion

Combining traditional pathology with high-throughput sequencing technology, this study identified *L. macrospora* as the causal agent of *Picea schrenkiana* needle blight and focused on clarifying the microecological dysbiosis of the needle microbiome driven by pathogen invasion. Beyond merely describing the succession of community structure, this study revealed the ecological process by which the cross-kingdom interaction network of needles undergoes structural disintegration from a healthy, diverse symbiotic steady state and ultimately experiences “cascade collapse” toward a pathogen-dominated, structurally simplified pathogenic network under the strong driving force of *L. macrospora*.

### 3.1. Pathogenicity and Ecological Strategy of L. macrospora

By fulfilling Koch’s postulates and integrating morphological and molecular data, we identified *L. macrospora* as the causal agent of *P. schrenkiana* needle blight. The characteristic symptoms on the infected needles and the morphological features of the pathogen are consistent with previous reports of *L. macrospora* on other coniferous species [8,19,20]. High-throughput sequencing provided critical insights into the pathogenic process. In strictly surface-sterilized healthy needles (YS-I), *L. macrospora* existed at a low relative abundance (2.56%), indicating its existence as an endophyte in *P. schrenkiana* needles, which is consistent with the study by Malin Elfstrand et al. [21]. Through high-throughput sequencing of vegetative buds from 518 unrelated Norway spruces (*Picea abies*), they found that pathogens like *L. macrospora*, *Rhizosphaera kalkhoffii*, and *Sydowia polyspora* can colonize asymptomatic tissues, existing as latent pathogens in seemingly healthy spruce needles and buds. Additionally, studies by Menkis [22], Nguyen [23], and Rajala [24,25] have shown that these pathogens are commonly present in the needle microbiome of healthy Norway spruce. However, as the disease progressed, both its relative and absolute abundances increased exponentially, establishing it as the absolute dominant species (>65%) in severely diseased tissues (YS-V). This transition from “latency” to “outbreak” is a typical infection strategy for opportunistic pathogens, which become aggressive when host’s physiological conditions change or the microbial ecosystem becomes imbalanced. Therefore, we proposed that the infection process of *L. macrospora* on *P. schrenkiana* needles includes an early asymptomatic colonization stage. During this stage, the pathogen resides as a latent endophyte within needle tissues. As needles age, environmental conditions become favorable, or host resistance declines, the pathogen transitions from the latent state to the pathogenic stage, proliferating rapidly and inducing typical symptoms of *P. schrenkiana* needle blight. The rapid proliferation and dominance of *L. macrospora* likely rely on specific pathogenic mechanisms. We hypothesize that, similar to other necrotrophic or hemi-biotrophic pathogens, *L. macrospora* may secrete cell wall-degrading enzymes (CWDEs) such as pectinases and cellulases to breach host cell barriers and access nutrients. Furthermore, the secretion of secondary metabolites or phytotoxins might be employed to suppress the host immune system and inhibit the growth of competing endophytes, thereby facilitating its rapid niche expansion [26,27].

Co-occurrence network analysis further elucidated the dynamic ecological role of *L. macrospora* throughout the infection process [28,29]. In the healthy needle community, *L. macrospora* exhibited significant negative correlations with numerous co-existing taxa, suggesting its population growth was effectively constrained by the native microbial community [30]. In moderately diseased tissues, the number of negative correlations involving *L. macrospora* increased, particularly with potential antagonists (e.g., *Sphingomonas*) [31] and original endophytic fungi (e.g., *Phaeococcomyces*), reflecting intensified niche competition between the pathogen and both potential antagonists and the established endophytic community. However, by the severe disease stage, the network connectivity of *L. macrospora* with other taxa decreased dramatically. This indicates that once it achieves dominance, the needle microbial community structure was simplified, the original micro-ecological balance was disrupted, and a new niche structure centered around the pathogen was established [32]. Synthesizing the network features across these three stages, the infection process of *L. macrospora* can be summarized as a successional progression from “community suppression” to “intensified niche competition”, and finally to the “establishment of a pathogen-centric dominant niche” [29,30].

### 3.2. Response of Microbial Community Diversity and Structure to Disease Progression

Fungal community diversity exhibited a trend of remaining stable initially followed by a sharp decline, while the structural changes in the bacterial community showed a pattern of first increasing and then decreasing. In moderately diseased tissues (YS-II), the structural diversity of the fungal community remained relatively high, whereas the bacterial community diversity reached its peak. This stage may correspond to the “community reorganization period” in the early stage of the disease [33,34]. On one hand, pathogen infection disturbed the composition of the original fungal community (e.g., the dominant genus *Phaeococcomyces*). On the other hand, the infection process and host physiological responses inevitably altered the phyllosphere microenvironment. The disruption of needle tissues likely created new ecological niches and released nutrients, facilitating colonization by a broader range of opportunistic fungi and bacteria. This structural shift explains the observed transient increase in microbial diversity, particularly in the bacterial community. Notably, this pattern is consistent with the findings of Li et al. [22], who reported similar community reorganization during rust disease development in apple leaves. This phenomenon has been interpreted in other studies as a potential “cry-for-help” strategy, wherein the host recruit beneficial microbes to alleviate stress, although the specific signaling mechanisms in this system remain to be explored.

At the severe disease stage (YS-V), the fungal community structure was markedly simplified and dominated by *L. macrospora* and a few saprophytes (e.g., *Limonomyces*). This resulted in a significant drop in fungal diversity, characteristic of a pathogen-dominated community collapse [16,32]. In contrast, while bacterial diversity decreased from its peak at the moderate stage, it remained higher than in the healthy group. This suggests that bacteria may assume more complex ecological roles in late-stage disease, potentially including members that act as collaborators with the pathogen, saprotrophs that exploit necrotic tissue, or even taxa that continue to exert antagonistic effects.

### 3.3. Ecological Roles and Bioindicator Potential of Core Microbial Taxa

*Phaeococcomyces* and *Rhizosphaera* exhibited highly consistent response pattern across the different disease severities, exhibiting typical characteristics of health-associated indicators. *Phaeococcomyces* was the dominant genus in healthy needles (relative abundance > 45%), but its abundance significantly decreased following infection by *L. macrospora*. This suggests that *Phaeococcomyces* may be a core endophyte that helps maintain the stability of the needle microbiome and suppress pathogens through mechanisms such as niche occupation, resource competition, secretion of antagonistic compounds, or induction of host defenses [35]. Similarly, the sharp decline in *Rhizosphaera* abundance with increasing disease severity indicates its high sensitivity to host health. Given the high sensitivity of *Phaeococcomyces* and *Rhizosphaera* to the disease, we propose that changes in their community abundance can be used as sensitive indicators to evaluate the degree of microbial dysbiosis in *P. schrenkiana* needles, providing an important microbiological basis for assessing host health status [36,37].

Conversely, the abundances of *Limonomyces* and certain *Frankia* taxa increased with disease severity, showing a typical “late-stage disease enrichment” pattern. Previous studies have shown that the *Limonomyces* species mostly consists of pathogenic fungi or endophytes of herbaceous and woody plants, but it can also persist as saprophytes in dead tissues under field conditions [38]. Additionally, some *Frankia* strains possess both nitrogen-fixing symbiotic capabilities and the ability to grow saprophytically on organic debris tissues [39]. In this study, *Limonomyces* and certain *Frankia* strains were significantly enriched in severely diseased tissues. We speculate that they may be secondary invaders or saprophytic commensals that undergo opportunistic colonization using the released nutrients after *L. macrospora* causes needle tissue necrosis [40,41].

The OTUs of *Sphingomonas* and *Methylobacterium* were significantly enriched during the moderate disease stage (YS-II). Previous studies have demonstrated that *Sphingomonas* and *Methylobacterium* are ubiquitously present in the plant phyllosphere microbiome, and certain strains possess the potential to promote host plant growth and enhance host stress tolerance [31,42]. Therefore, we speculate that the observed enrichment in the moderate infection stage may be related to the host’s defense response to pathogen invasion. This phenomenon shares similar ecological characteristics with the “cry for help” hypothesis proposed in the rhizosphere microbiome, where stressed plants may regulate physiological metabolism to enrich beneficial microorganisms [43,44]. The temporary increase in *Sphingomonas* and *Methylobacterium* in the moderate infection stage in this study suggests that they may play a role as “key transitional taxa” in community succession. Their potential interactions with *L. macrospora* may affect the dynamic balance of the community, although their specific ecological functions and driving mechanisms require further verification.

### 3.4. Microbial Co-Occurrence Network: From Community Homeostasis to Pathogen Dominance

Co-occurrence network analysis revealed the systemic reorganization of microbial interaction patterns during disease development from a community structure perspective [45]. The network of healthy needles (YS-I), though having fewer nodes, exhibited high overall connectivity with relatively balanced positive and negative correlations, indicating a compact and stable community structure [32]. At this stage, *L. macrospora* did not exhibit characteristics of a hub or keystone species, suggesting that in the healthy state, it does not dictate community assembly but exists as a peripheral member or latent endophyte with low abundance [21,46], consistent with the characteristics of the latent phase. In the moderate disease stage (YS-II), network complexity and the proportion of negative correlations peaked, reflecting the emergence of numerous new nodes and interactions and a shift from a compact to a highly restructured community [16]. At this stage, various taxa, including opportunistic bacteria and non-dominant fungi, were drawn into more complex relationships. The increased number of negative correlations involving *L. macrospora* highlights that niche competition and antagonistic interactions with native endophytes and potential bacterial antagonists were most prominent, lending the network a feature of enhanced competition [47].

When the disease advanced to the severe disease stage (YS-V), network complexity declined significantly, with a reduction in both nodes and edges, while the proportion of positive correlations increased. This is likely because the community structure was simplified into a co-occurring consortium of a few dominant taxa [32,48]. At this point, *L. macrospora* and a few saprotrophic fungi formed the new core of the network. We infer that in this final stage, the suppressive capacity of the original community against the pathogen was significantly diminished. The needle microbiome transitioned into a community structure geared towards the co-exploitation and decomposition of host tissues by the pathogen and its associated saprophytic/commensal taxa. Ecologically, this can be viewed as a “functional collapse” or dysbiosis of the community, driven by the pathogen [40,49]. Although correlation-based networks do not directly prove mechanistic interactions, the systematic changes in network structure across these three stages provide a compelling conceptual model for the ecological process of “community stability breakdown → intensified competition → pathogen dominance”. This drastic shift from a high-competition state (YS-II) to a collapsed, pathogen-dominated state (YS-V) suggests the existence of an ecological tipping point. We propose that once the abundance of *L. macrospora* exceeds a critical threshold, it triggers a self-reinforcing feedback loop: the pathogen suppresses keystone taxa (e.g., *Phaeococcomyces*), leading to a breakdown in community resilience. This loss of ‘microbial colonization resistance’ allows the pathogen to grow unchecked, driving the ecosystem past a point of no return toward irreversible dysbiosis [50,51].

## 4. Conclusions

By integrating ITS and 16S rRNA amplicon sequencing, this study resolved the successional trajectory of the needle microbiome across different infection gradients of *P. schrenkiana* needle blight caused by *L. macrospora*. The results reveal the ecological dynamics of *L. macrospora* within the needle microenvironment: it exists as an “endophyte” in healthy needles, subsequently expands and reshapes community structure as the disease progresses, and ultimately evolves into a dominant pathogen causing microecological dysbiosis. Based on these findings, we propose a conceptual model of “endophyte-expansion-dominance” to provide a framework for understanding the ecological dynamics of this disease. Looking forward, it is imperative to combine metagenomics, metatranscriptomics, and targeted isolation-reinoculation experiments to further dissect the mechanistic interactions between *L. macrospora* and core microbiome members, thereby validating the key functional and molecular pathways driving its transition from endophyte to pathogen.

## 5. Materials and Methods

### 5.1. Experimental Design and Sample Collection

The study was conducted in Tablehet Mongol Ethnic Township, Wusu City, Tacheng Prefecture, Xinjiang Uygur Autonomous Region, China (84°7′35″ E, 44°10′48″ N). The region is characterized by a temperate continental climate with an annual sunshine duration of 4444 h, an extreme minimum temperature of −37.5 °C, and an extreme maximum temperature of 42.2 °C. The dominant tree species in this area is *P. schrenkiana*. Three standard plots were established within a representative *P. schrenkiana* stand. A total of 50 trees exhibiting similar growth vigor and disease incidence were selected for sampling. All samples were placed in sterile bags and immediately transported to the laboratory on ice. Needle samples were classified into five disease severity levels according to the percentage of lesion area (Table 1).

To exclude the interference of epiphytic microorganisms, needles intended for pathogen isolation and amplicon sequencing underwent a strict surface sterilization protocol involving immersion in 75% ethanol for 1 min, rinsing five times with sterile water, immersion in 1% sodium hypochlorite (NaClO) solution for 3 min, and another five times rinse with sterile water. The last rinse water was spread on potato dextrose agar (PDA) medium to confirm the absence of epiphytic contaminants on the sample surface [52,53]. Samples for amplicon sequencing were stored at −80 °C, while those for pathogen isolation were preserved at 4 °C. To clearly reveal the successional dynamics of microbial community structure during disease progression and ensure significant differentiation among groups, needle samples representing three key stages (YS-I for healthy, YS-II for moderately infected, and YS-V for severely infected) were specifically selected for subsequent determination and analysis.

### 5.2. Pathogen Isolation, Purification, and Pathogenicity Test

Isolates were obtained from surface-sterilized, symptomatic needles using a tissue isolation method under sterile conditions in a laminar flow hood. The tissue fragments were placed on PDA medium and incubated at 25 °C in the dark. Hyphal tips from the margins of emerging colonies were transferred to fresh PDA slants for purification.

To fulfill Koch’s postulates, pathogenicity tests were performed on healthy, potted *P. schrenkiana* seedlings. Needles were first surface-wiped with 75% ethanol. Mycelial plugs (6 mm diameter) were excised from the periphery of an actively growing colony of the fungal isolate and used for inoculation via three methods (*n* = 10 replicates for each method): wounding (pricking with a sterile needle), heat-scarification, and non-wounding (direct contact). The inoculation sites were then wrapped with sterile gauze and enclosed in sterile plastic bags to maintain high humidity. For the control group, a sterile PDA medium was used instead of mycelial plugs [54]. After 48 h, the bags and gauze were removed, and the seedlings were maintained under same conditions. The needles were monitored every three days for symptom development. Once symptoms appeared, they were observed daily to record disease progression. The fungus was subsequently re-isolated from the symptomatic lesions, purified, and preserved to confirm its role as the causal agent.

### 5.3. Pathogen Identification

Infected needles were transported to the laboratory, and free-hand sections were prepared. Morphological characteristics of pathogen fruiting bodies on the needle surface were observed under an optical microscope (XSP-12CA) and electron microscope, and the size and structure of asci and ascospores were measured and recorded. Meanwhile, the colony morphology, color, and growth characteristics of purified pathogens on PDA medium were documented. For molecular identification, fungal genomic DNA was extracted from mycelia cultured on PDA using the E.Z.N.A.™ Fungal DNA Kit (Omega Bio-Tek, Darmstadt, Germany) according to the manufacturer’s protocol [55]. The Internal Transcribed Spacer (ITS) region, as well as the AF462441 and 58S gene regions, were amplified and sequenced using the primer pairs ITS1/ITS4, AF462441-1-F/AF462441-1-R, and 58S-F/58S-R, respectively. The 50 μL PCR reaction mixture contained 10 µL of 5× FastPfu Buffer, 2 µL of 2.5 mM dNTPs, 1 µL of each forward and reverse primer (5 µM), 0.5 µL of FastPfu Polymerase, 10 ng of template DNA, and was brought to volume with 14.5 µL of ddH_2_O. The thermocycling conditions were: initial denaturation at 95 °C for 5 min; followed by 29 cycles (for ITS1/ITS4) or 30 cycles (for AF462441 and 58S fragments) of denaturation at 95 °C for 30 s, annealing at 55 °C for 30 s, and extension at 72 °C for 45 s; and a final extension at 72 °C for 10 min. The quality of PCR products was assessed by agarose gel electrophoresis prior to sequencing. The resulting sequences were assembled and subjected to a BLASTn search against the NCBI database. All sequences have been deposited in NCBI GenBank under the accession numbers PV544969, PV544970, and PV544971. A phylogenetic tree was constructed based on the concatenated sequences of the ITS, AF462441, and 58S genes using the Maximum Likelihood method in MEGA 11 [56].

### 5.4. Amplicon Sequencing and Pathogen Quantification

Following the surface sterilization protocol described in Section 5.2, total genomic DNA was extracted from the needle samples using the E.Z.N.A.^®^ Soil DNA Kit (Omega Bio-Tek, Norcross, GA, USA) following the manufacturer’s instructions. DNA purity and concentration were assessed using a NanoDrop 2000 spectrophotometer (Thermo Fisher Scientific, Waltham, MA, USA), and DNA integrity was verified by 1% agarose gel electrophoresis.

The ITS1-ITS2 region of fungal was amplified using primers ITS1F (5′-CTTGGTCATTTAGAGGAAGTAA-3′) and ITS2R (5′-GCTGCGTTCTTCATCGATGC-3′) [57]. The V3-V4 hypervariable region of the bacterial 16S rRNA gene was amplified using primers 341F (5′-CCTAYGGGRBGCASCAG-3′) and 806R (5′-GGACTACNNGGGTATCTAAT-3′). Each primer was barcoded with an 8-base tag sequence to distinguish samples. The 20 μL PCR reaction consisted of 4 μL of 5× FastPfu Buffer, 2 μL of 2.5 mM dNTPs, 0.8 μL of each primer (5 μM), 0.4 μL of FastPfu Polymerase, 10 ng of template DNA, and was supplemented with sterile water to the final volume. PCR amplification was performed under the following conditions: initial denaturation at 94 °C for 4 min; followed by 25 cycles of 94 °C for 30 s, 55 °C for 30 s, and 72 °C for 1 min; and a final extension at 72 °C for 10 min. Amplicons were purified using the AxyPrep DNA Gel Extraction Kit (Axygen Biosciences, San Jose, CA, USA) and pooled to construct a sequencing library, which was then subjected to paired-end sequencing on an Illumina MiSeq PE300 platform [58].

Raw sequencing data were processed through quality control and chimera removal to obtain effective sequences. The resulting high-quality sequences were clustered into Operational Taxonomic Units (OTUs) at a 97% similarity threshold using UPARSE (version 7.1). Taxonomic annotation was performed using the UCLUST algorithm against the GTDB-16S database (r220) for bacteria and the NCBI ITS database (downloaded in 2025) for fungi, with a confidence threshold of 80%. The raw sequencing data generated in this study have been deposited in the NCBI Sequence Read Archive (SRA) under the BioProject accession number PRJNA1400240.

Absolute quantification of the pathogen *L. macrospora* was performed using quantitative PCR (qPCR) with primers ITS1F and ITS2R. The 30 μL qPCR reaction mixture included 1.5 μL of qPCR Mix, 0.5 μL of each primer (10 μM), 2 μL of template DNA, and 25.5 μL of ddH_2_O. The qPCR program was: 95 °C for 3 min; followed by 40 cycles of 95 °C for 15 s, 57 °C for 20 s, and 72 °C for 20 s; and a final extension at 72 °C for 5 min. A standard curve was generated using a 10-fold serial dilution of a plasmid containing the target gene fragment, with sterile water as a negative control. The standard curve exhibited an amplification efficiency of 95.7% and a correlation coefficient (R^2^) of 0.991. Data were processed and calculated in Origin 2025b.

### 5.5. Data and Statistical Analysis

Alpha diversity indices were calculated using mothur (v1.48.0,). To assess beta diversity, Principal Co-ordinates Analysis (PCoA) was performed based on Bray–Curtis dissimilarity matrices using the “vegan” package (v2.6-0) in R. The influence of pathogen infection on the needle microbial community composition was evaluated using Permutational Multivariate Analysis of Variance (PERMANOVA) via the “adonis” function within the “vegan” package. Venn diagrams were generated using the online tool Draw Venn Diagram. Differentially abundant species were identified using the Wilcoxon rank-sum test, and a heatmap at the species level was generated using the “pheatmap” package in R. An inter-kingdom microbial co-occurrence network was constructed for fungal and bacterial communities at the OTU level across the YS-I, YS-II, and YS-V groups. OTUs with a relative abundance below 0.01% were filtered out. The recombinant plasmid containing the target gene was purified and quantified. Its concentration was converted into copy number based on the molecular weight. A standard curve was generated using 10-fold serial dilutions of the plasmid, ranging from 10^−2^ to 10^−6^ copies/μL. The absolute quantity of *L. macrospora* in each sample was calculated by interpolating the cycle threshold (Ct) values against the standard curve (R^2^ > 0.99). A correlation matrix was then generated using the “psych” package in R. Finally, network topological parameters were calculated, and the network was visualized using Gephi (v0.9.2).

## Figures and Tables

**Table 1:** Disease severity classification for *P. schrenkiana* needle blight.

Disease Level	Grading Criteria	Grading Criteria
I	healthy needles with no visible lesions	0
II	Lesion area < 25%	1
III	25% < Lesion area < 50%	2
IV	50%< Lesion area < 75%	3
V	Lesion area > 75%	4

## Data Availability

The data that support the findings of this study are available from the corresponding authors upon reasonable request. The datasets generated and/or analyzed during the current study are available in the NCBI repository, PRJNA1180918.
